# Giant left anterior descending artery aneurysm in a patient with active systemic lupus erythematosus: a case report

**DOI:** 10.1186/s13019-021-01725-2

**Published:** 2021-11-21

**Authors:** Zipeng Yao, Yanhong Long, Zheng Zong, Lin Wang

**Affiliations:** 1 Department of Cardiology, The First People’s Hospital of Tianmen, Tianmen, 431700 China; 2 Department of Cardiology, The First People’s Hospital of Jining, Jining, 272000 China; 3 Department of Cardiovascular Center, The First Hospital of Jilin University, No.71 Xinmin Street, Changchun, 130021 China

**Keywords:** Systemic lupus erythematosus, Coronary artery aneurysm, Coronary angiography

## Abstract

**Background:**

Although not common, coronary artery aneurysms (CAAs) can develop to over 8 mm in diameter to become giant CAAs. In the context of systemic lupus erythematosus (SLE), autoantibody- and immune complex-mediated atherosclerosis is believed to be the most prevalent cause of aneurysm.

**Case presentation:**

We report the case of a 53-year-old female SLE patient who presented to our hospital with radiating chest pain. Coronary angiography revealed a giant aneurysm in the middle segment of the left anterior descending artery (LAD) and distal subtotal occlusion in the left circumflex artery (LCX). Laboratory testing also identified risk factors such as an abnormal pulmonary enzyme profile, dyslipidemia, and nephritis parameters.To prevent thromboembolism, anticoagulation and antiplatelet therapy were administered. In addition, one stent was implanted at the distal end of the LCX and repeated coronary angiography verified restoration of TIMI grade III flow.The patient was discharged with resolved chest pain. During 6 months of follow-up, the patient is in good health.

**Conclusions:**

Our case study, together with 16 recent comparable reports, emphasizes the need for coronary aneurysm screening in SLE patients. It is necessary that thromboembolism, anticoagulation and antiplatelet therapy were administered for CAA.

## Introduction

While atherosclerosis is the most prevalent cause of coronary artery aneurysm (CAA) and atherosclerosis is a complication of systemic lupus erythematosus (SLE), CAA formation in SLE patients is rare [1, 2]. The prognosis and treatment of CAA are significantly impacted by complications such as thrombosis, distal occlusion, and aneurysm rupture [1]. Currently, however, there is no formal guide for the optimal treatment of CAAs in patients with SLE, and thus, validated clinical studies are urgently needed. Here, we present the a case study of a patient with active SLE who presented with a giant CAA.

## Case description

**Fig. 1:**
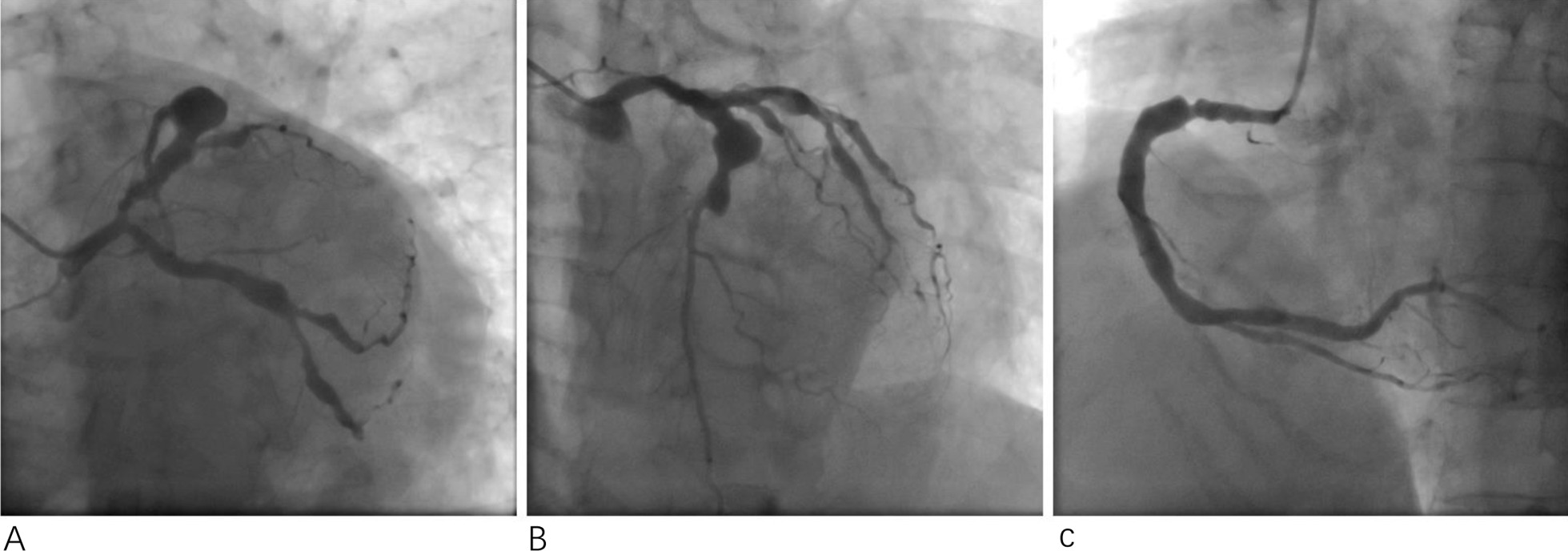
Coronary angiography: A giant aneurysm was observed in the middle of the left anterior descending artery (LAD) (**a**). In the left circumflex artery (LCX), a proximal cystic-like dilatation and distal subtotal occlusion with TIMI grade I flow were observed (**b**). Different degrees of dilatation were detected in the middle of the right coronary artery (RCA) (**c**)

A 53-year-old female patient was admitted to our hospital due to chest pain radiating to the throat. This symptom had developed 1 month previously, and episodes lasted approximately 30 min before temporarily relief was achieved by oral nitroglycerin administration. The patient had been diagnosed with SLE at the age of 33 years and treated with oral corticosteroids (12.5 mg/day prednisone), leflunomide, and hydroxychloroquine thereafter. However, no records of periodic interviews or comprehensive documents were available. She denied a history of smoking or drinking Thirteen years after the diagnosis of SLE, she was diagnosed with hypertension with a blood pressure of 180/90 mmHg and began treatment with oral nitrendipine at a dose of 10 mg/day. Physical examination on the current admission revealed a heart rate of 88 beats per minute, a respiration rate of 18 breaths per minute, and a blood pressure of 118/62 mmHg with regular heart rhythm and no positive signs, suggesting the hypertension was well controlled by the medication. An electrocardiogram showed normal sinus rhythm, right axis deviation, and no ST-T abnormality, but did reveal fractional shortening by 29%, an ejection fraction of 56%, a small amount of pericardial effusion, and a decreased amplitude of the ventricular septum and left ventricular inferior wall, indicating heart dysfunction. Coronary angiography then revealed a giant aneurysm in the middle segment of the left anterior descending artery (LAD; Fig. 1a and b). In the left circumflex artery (LCX), proximal cystic-like dilatation and distal subtotal occlusion with TIMI grade I flow were observed (Fig. 1b). In addition, varying degrees of dilatation were detected in the middle segment of the right coronary artery (RCA; Fig. 1c).

Laboratory examination results for creatine kinase-muscle/brain (2.00 ng/ml, normal range 0–4.3 ng/ml) showed that oral nitroglycerin was effective in the early stage. However, elevated serum levels of myoglobin (155.00 ng/ml, normal range 0–107 ng/ml), cardiac troponin I (0.62 ng/ml, normal range 0–0.05 ng/ml), and aspartate aminotransferase (75.4 U/L, normal range 13–35 U/L) indicated irreversible myocardial infraction. In the meantime, the presence of active SLE was verified by the autoimmune profile results, including normal complement C3 (0.82 g/L, normal range 0.7–1.4 g/L) and complement C4 (0.27 g/L, normal range 0.1–0.4 g/L) levels as well as positive detection of anti-dsDNA (IIF) at 1:10, homozygous antinuclear antibodies (ANA) at 1:100, and nuclear membrane type ANA at 1:320 along with a high C-reactive protein concentration (16.20 mg/L, normal range 0–3.5 mg/L) and high erythrocyte sedimentation rate (44 mm/1 h, normal range 0–20 mm/1 h). Two risk factors for both SLE and cardiovascular disease also were identified in this case. First, renal parameters were abnormal with a 1 + positive urine protein, positive urinary nitrite, and elevated levels of urea nitrogen (20.59 mmol/L, normal range 2.6–7.5 mmol/L) and creatinine (167.8 µmol/L, normal range 41–73 µmol/L). Additionally, elevated lipid levels including a cholesterol level of 7.17 mmol/L (normal range 2.6–6.0 mmol/L) and triglyceride level of 2.86 mmol/L (normal range 0.28–1.8 mmol/L) implied dyslipidemia. Finally, The patient had been diagnosed with SLE, CAA,hypertension and acute coronary syndrome.

### Therapeutic intervention

**Fig. 2:**
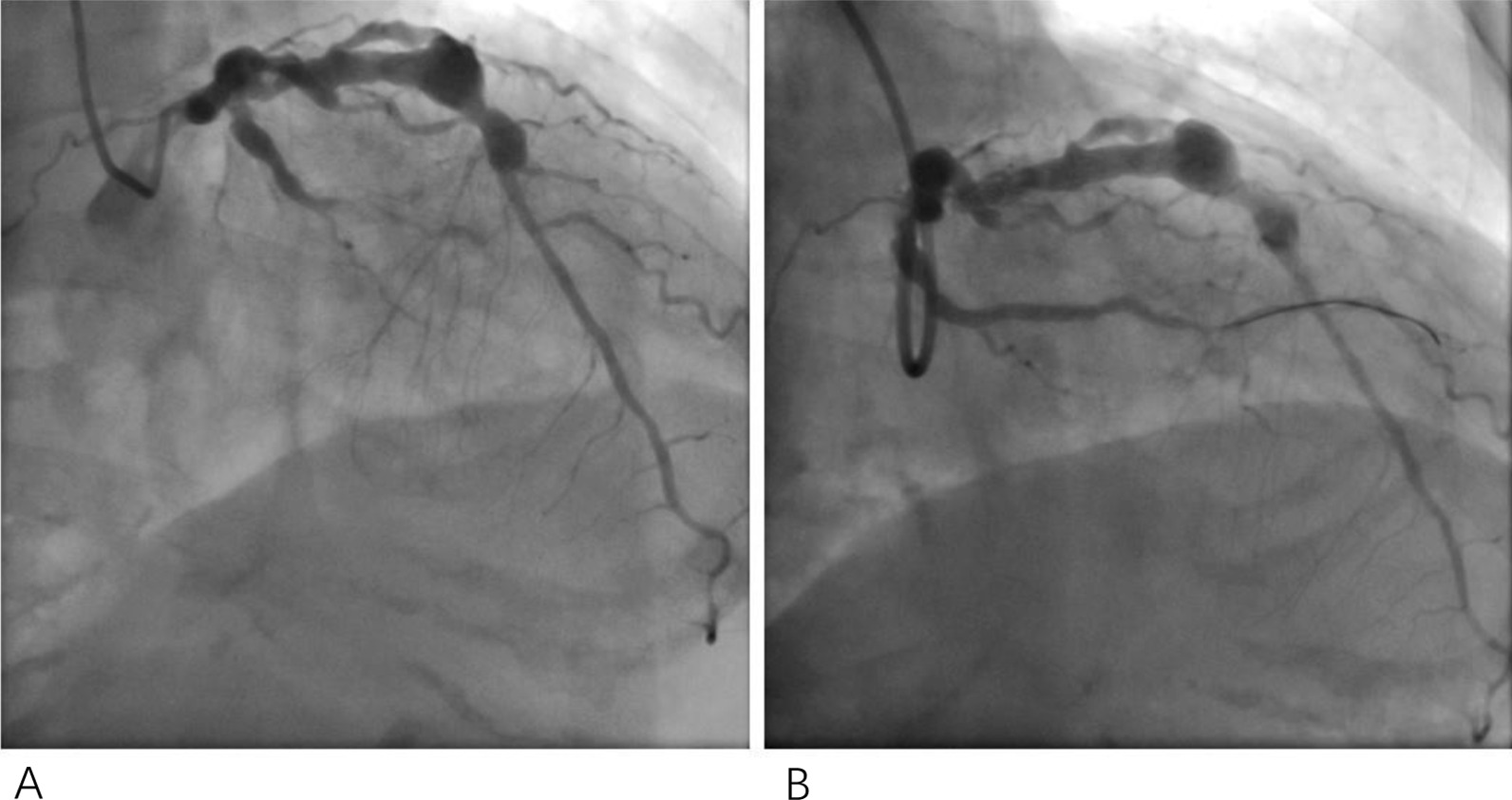
One stent was implanted at the distal end of the LCX (**a**). Repeated coronary angiography revealed that the occlusion was recanalized and TIMI grade III flow was restored (**b**)

Surgical treatment of giant aneurysms is considered to be a major trauma, so patients choose interventional therapy.At the same time, surgical treatment is our second option. To prevent thromboembolic complications and further myocardial infarction, anticoagulation (low-molecular-weight calcium heparins, 5,000 U subcutaneously every 12 h) and antiplatelet therapy (oral aspirin 100 mg/day and oral clopidogrel hydrogen sulfate 75 mg/day) were administered. At the same time, one stent was implanted at the distal end of the LCX to effectively limit further expansion of the affected coronary segments (Fig. 2a). Repeated coronary angiography revealed that the occlusion was recanalized and TIMI grade III flow was restored (Fig. 2b). Finally, the patient was discharged with resolved chest pain. New oral anticoagulant (rivaroxaban) and dual antiplatelet therapy recommended at discharge. Furthermore patient has ectasia and a significant stenosis in proximal RCA.Coronary angiography was recommended after 1 month, but the patient refused..After 1 month, they were treated with rivaroxaban and aspirin. During 6 months of follow-up, the patient is in good health.

## Discussion

In this study, we reported an active SLE patient with a giant LAD aneurysm. CAA is an irreversible coronary vascular dilatation that occasionally grows to become considered giant with a diameter of more than 8 mm. In early studies, the prevalence of giant CAA was only 0.02% among all CAA cases, and the pathogenesis generally involves other diseases, including SLE [1]. In SLE-related CAA, autoantibody- and immune complex-mediated arterial media destruction and arterial wall thinning are believed to result in the progressive dilatation of the affected arterial segments [2]. However, long-term use of immunosuppressive drugs may also accelerate coronary artery disease [3]. Thus, it is not possible to completely exclude the effects of steroid therapy on CAA pathogenesis. Is there any role for modifications in medical therapy of SLE for treating this disease? At present, there seems to be no definite answer.Perhaps particular disease-modifying anti-rheumatic drugs might have different effects.

As summarized in Table 1, 16 cases of coronary aneurysm in SLE patients have been reported recently. The average age of these patients was 38 years, and most were women (14 cases). The duration of SLE varied from 1–42 years. Most cases (9 cases) had symptoms of ischemic heart disease caused by embolization or stenosis of the coronary artery. Moreover, more than half (9) of cases had atherosclerosis risk factors in addition to SLE (6 cases with hypertension, 2 cases with hyperlipidemia, and 1 case with a smoking history). It should also be noted that the ANA titer was positive in half of the cases, and only two cases were antiphospholipid antibody positive [6, 7]. Similarly, several typical common features were identified in our case. First, 13 years before the patient was diagnosed with hypertension, the diagnosis of SLE was made. Although anti-lupus medication was never discontinued, the ANA test indicated that the SLE was in the active stage when the patient presented to us. Second, although the patient’s hypertension had been controlled, the enzyme profile implied irreversible myocardial infarction. Furthermore, two other clinical markers, hyperlipidemia and nephritis, were also detected.

**Table.1:** Summary of reports of SLE patients with coronary aneurysm expansion

	Age, years	Sex	Duration of SLE, years	Location (s)	Symptom (s)	Risk factor (s)	ANA	APLAs
Present case	53	F	20	LAD, LCX, RCA	CP	H	1:320	none
Geiser [4]	29	F	6	LAD, LCX, RCA	Confusion	H	ND	ND
Ha [5]	21	F	1	RCA	PT	ND	ND	ND
Suzuki [6]	31	F	20	LCX, RCA	None	None	–	+
Hirata [7]	34	F	12	LAD, RCA	CP	None	ND	+
Monigari [8]	30	F	3	LAD	None	None	ND	ND
Matayoshi [9]	29	F	6	LAD, LCX, RCA	None	None	1:160	–
Wilson [10]	25	M	8	LAD	CP	ND	1:2560	–
	44	F	11	LAD	CP	H	1:1280	ND
Caracciolo [11]	22	F	4	LAD	CP	SH	1:320	–
Uchida [12]	55	M	42	LCX, RCA	CP	H, HL	1:80	ND
Yoshikai [13]	49	F	10	RCA	AMI	HL	1:640	ND
Anna [14]	65	F	7	RCA	CP	H	1:300	ND
Famularo [15]	32	F	ND	LAD, LCX, RCA	None	H	ND	ND
Nobrega [16]	26	F	12	LAD, RCA	None	ND	ND	–
Nagao [17]	62	F	20	LAD	CP	ND	ND	ND

LAD: left anterior descending artery, LCX: left circumflex branch, RCA: right coronary artery, AMI: acute myocardial infarction, CP: chest pain, DR: dyspneic respiration, H: hypertension, HL: hyperlipidemia, ND: no description provided, SH: smoking history, PT: pericardial tamponade, ANA: antinuclear antibody, APLAs: antiphospholipid antibodies

In this case, as in most other cases of SLE patients with coronary artery stenosis, calcium heparins, aspirin, and clopidogrel hydrogen sulfate were used to prevent thromboembolism [16, 18]. Aneurysm rupture is difficult to predict and can be catastrophic if it occurs. While the outcome of percutaneous coronary intervention in patients with autoimmune disease remains uncertain [19], coronary angiography verified that stent implantation restored the blood supply in our case. However, for most SLE patients with coronary aneurysms, there are currently no official guidelines for the best treatment strategies. Therefore, SLE patients with risk factors such as hypertension, hyperlipidemia, and nephritis, should be offered CAA screening to avoid delays in diagnosis and treatment. At the same time, more clinical trials are required to verify the appropriate therapy for coronary dilatation in the context of SLE, particularly for large aneurysms.

## Conclusion

Our case study, together with 16 recent comparable reports, emphasizes the need for coronary aneurysm screening in SLE patients. It is necessary that thromboembolism, anticoagulation and antiplatelet therapy were administered for CAA.

## Data Availability

This case is true and effective.

## Abbreviations

CAA: Coronary artery aneurysm
SLE: Systemic lupus erythematosus
LAD: Left anterior descending artery
LCX: Left circumflex artery
TIMI: Thrombolysis in myocardial infarction
ANA: Antinuclear antibodies
